# Supplementary material for: Elevating levels of neuronal MCU in the hippocampus enhances mitochondrial calcium uptake and respiratory efficiency proportional to demand
Source: bioRxiv. 2026 Apr 16:2026.04.13.718264. Preprint. [Version 1] doi: 10.64898/2026.04.13.718264 (PMC13105083; doi:10.64898/2026.04.13.718264)
Supplement: Supplement 2 [file media-2.pdf]

Supplemental Table

Cohort	Lane I	Strain	Sex	Group	Mouse ID	Respirati	Calcium	Swelling	MCU OE WI	MICU1 W	Total Oxphos W	TEM	Notes
Cohort 1	H1	Am2-eGFP	Male	GFP	G823	O2k	CRC	mPTP	MCU OE				
Cohort 1	H2	TDtom	Male	MCU	Td490	O2k	CRC	mPTP	MCU OE				
Cohort 1	H3	TDtom	Female	GFP	Td516	O2k	CRC		MCU OE				
Cohort 1	H4	TDtom	Female	MCU	Td515	O2k	CRC		MCU OE				
Cohort 1	H5	TDtom	Male	MCU	Td513		CRC		MCU OE				
Cohort 1	H6	C57bl6 /N	Female	WT	M245		CRC		MCU OE				Cohort 1 H5, H6 - Pair was excluded from CRC experiments and MCU OE quantification because unblinding revealed MCU OE and WT pairing, not a GFP-expressing CTL
Cohort 1	H7	C57bl6 /N	Female	GFP	M226	O2k	CRC		MCU OE				
Cohort 1	H8	C57bl6 /N	Female	MCU	M227	O2k	CRC		MCU OE				
Cohort 2	H1	C57bl6 /N	Female	GFP	M229	O2k	CRC		MCU OE				Cohort 2 H1, H2 - Pair excluded from O2k experiments because state 1 negative OCR, likely indicating poor instrument calibration
Cohort 2	H2	C57bl6 /N	Female	MCU	M228	O2k	CRC		MCU OE				
Cohort 2	H3	C57bl6 /N	Female	MCU	M237	O2k	CRC		MCU OE				Cohort 2 H3, H4 - Pair excluded from CRC experiments due to failed CRC assay and not enough mitochondria to repeat the experiment
Cohort 2	H4	C57bl6 /N	Female	GFP	M238	O2k	CRC		MCU OE				
Cohort 2	H5	C57bl6 /N	Male	GFP	M239	O2k	CRC	mPTP	MCU OE				Cohort 2 H5, H6 - Pair excluded from CRC quantification because calcium uptake rates were ≥ 2 SD from sample population
Cohort 2	H6	C57bl6 /N	Male	MCU	M240	O2k	CRC	mPTP	MCU OE				
Cohort 3	H1	C57bl6 /J	Female	GFP	G4 -3	O2k	CRC		MCU OE	MICU1	Total Oxphos		
Cohort 3	H2	C57bl6 /J	Female	MCU	G4 -4	O2k	CRC		MCU OE	MICU1	Total Oxphos		
Cohort 3	H3	TDtom	Female	GFP	Td565	O2k	CRC		MCU OE	MICU1	Total Oxphos		
Cohort 3	H4	TDtom	Female	MCU	G5-3	O2k	CRC		MCU OE	MICU1	Total Oxphos		
Cohort 3	H5	C57bl6 /J	Female	GFP	B008				MCU OE	MICU1	Total Oxphos		
Cohort 3	H6	C57bl6 /J	Female	MCU	B005				MCU OE	MICU1	Total Oxphos		
Cohort 3	H7	C57bl6 /J	Female	MCU	B007				MCU OE	MICU1	Total Oxphos		
Cohort 3	H8	C57bl6 /J	Female	GFP	B006				MCU OE	MICU1	Total Oxphos		
Cohort 4	H1	C57bl6 /J	Male	GFP	G3-2	O2k	CRC		MCU OE				
Cohort 4	H2	C57bl6 /J	Male	MCU	G3-1	O2k	CRC		MCU OE				
Cohort 4	H3	C57bl6 /J	Female	MCU	G4-2	O2k	CRC		MCU OE				
Cohort 4	H4	C57bl6 /J	Female	GFP	G4-1	O2k	CRC		MCU OE				
Cohort 4	H5	C57bl6 /J	Female	MCU	G12-1				MCU OE				
Cohort 4	H6	C57bl6 /J	Female	GFP	G12-2				MCU OE				
Cohort 5	H1	C57bl6 /J	Female	GFP	B015			mPTP	MCU OE			TEM	Cohort 5 H1, H2 - Pair excluded from MCU OE quantification due to low recovery and no response to mPTP assay
Cohort 5	H2	C57bl6 /J	Female	MCU	B016			mPTP	MCU OE			TEM	
Cohort 5	H3	C57bl6 /J	Female	MCU	B017			mPTP	MCU OE			TEM	
Cohort 5	H4	C57bl6 /J	Female	GFP	B018			mPTP	MCU OE			TEM	

Cohort	Lane I	Strain	Sex	Group	Mouse ID	Respirati	Calcium	Swelling	MCU OE WI	MICU1 W	Total Oxphos W	TEM	Notes
Cohort 6	H1	C57bl6 /J	Female	GFP	B036			mPTP	MCU OE		Total Oxphos	TEM	
Cohort 6	H2	C57bl6 /J	Female	MCU	B026			mPTP	MCU OE		Total Oxphos	TEM	
Cohort 6	H3	C57bl6 /J	Female	GFP	B035			mPTP	MCU OE		Total Oxphos	TEM	
Cohort 6	H4	C57bl6 /J	Female	MCU	B038			mPTP	MCU OE		Total Oxphos	TEM	
Cohort 6	H5	C57bl6 /J	Female	MCU	SS5			mPTP	MCU OE		Total Oxphos	TEM	
Cohort 6	H6	C57bl6 /J	Female	GFP	SS4			mPTP	MCU OE		Total Oxphos	TEM	
Cohort 6	H7	C57bl6 /J	Female	MCU	SS7			mPTP	MCU OE		Total Oxphos	TEM	Cohort 6 H7, H8 - Pair excluded from total oxphos quantification due to gel/image artifact at CI and CII band
Cohort 6	H8	C57bl6 /J	Female	GFP	SS6			mPTP	MCU OE		Total Oxphos	TEM	
Cohort 7	H1	C57bl6 /J	Male	MCU	B070			mPTP		MICU1	Total Oxphos	TEM	Cohort 7 H1, H2 - Pair excluded from mPTP quantification due to failed mPTP assay, or no increase in extramitochondrial calcium release
Cohort 7	H2	C57bl6 /J	Male	GFP	B071			mPTP		MICU1	Total Oxphos	TEM	
Cohort 7	H3	C57bl6 /J	Male	GFP	B074			mPTP		MICU1	Total Oxphos	TEM	
Cohort 7	H4	C57bl6 /J	Male	MCU	B072			mPTP		MICU1	Total Oxphos	TEM	
Cohort 7	H5	C57bl6 /J	Female	MCU	B080			mPTP		MICU1	Total Oxphos	TEM	
Cohort 7	H6	C57bl6 /J	Female	GFP	B081			mPTP		MICU1	Total Oxphos	TEM	
Cohort 7	H7	C57bl6 /J	Female	MCU	B085			mPTP		MICU1	Total Oxphos	TEM	
Cohort 7	H8	C57bl6 /J	Female	GFP	B086			mPTP		MICU1	Total Oxphos	TEM	
